# Combining phage display with SMRTbell next-generation sequencing for the rapid discovery of functional scFv fragments

**DOI:** 10.1080/19420862.2020.1864084

**Published:** 2020-12-31

**Authors:** Francesco Nannini, Lenart Senicar, Farhaan Parekh, Khai J. Kong, Alexander Kinna, Reyisa Bughda, James Sillibourne, Xihao Hu, Biao Ma, Yuchen Bai, Mathieu Ferrari, Martin A. Pule, Shimobi C. Onuoha

**Affiliations:** a Cancer Institute, University College London, London, UK; b Autolus Therapeutics, London, UK; c GV20 Therapeutics LLC, Cambridge, MA, USA

**Keywords:** Antibody discovery, rat immune libraries, phage display, long-read sequencing, scFv antibody, affinity modulation

## Abstract

Phage display technology in combination with next-generation sequencing (NGS) currently is a state-of-the-art method for the enrichment and isolation of monoclonal antibodies from diverse libraries. However, the current NGS methods employed for sequencing phage display libraries are limited by the short contiguous read lengths associated with second-generation sequencing platforms. Consequently, the identification of antibody sequences has conventionally been restricted to individual antibody domains or to the analysis of single domain binding moieties such as camelid VHH or cartilaginous fish IgNAR antibodies. In this study, we report the application of third-generation sequencing to address this limitation. We used single molecule real time (SMRT) sequencing coupled with hairpin adaptor loop ligation to facilitate the accurate interrogation of full-length single-chain Fv (scFv) libraries. Our method facilitated the rapid isolation and testing of scFv antibodies enriched from phage display libraries within days following panning. Two libraries against CD160 and CD123 were panned and monitored by NGS. Analysis of NGS antibody data sets led to the isolation of several functional scFv antibodies that were not identified by conventional panning and screening strategies. Our approach, which combines phage display selection of immune libraries with the full-length interrogation of scFv fragments, is an easy method to discover functional antibodies, with a range of affinities and biophysical characteristics.

## Introduction

Display technologies, such as phage, yeast and ribosome display are important tools in the identification of therapeutic antibodies.^1^ In particular, phage display is widely used at several stages of the drug discovery process. Phage library diversity can be derived from naïve human B cells,^2^ synthetic engineered antibody sequences^3^ or from B cell-rich tissue of immunized animals;^4^ libraries generated by the latter method are often referred to as immune phage display libraries. Immune phage display offers several advantages over naïve or synthetic display approaches, increasing the likelihood of isolating stable, high-affinity specific antibodies due to pre-enrichment and *in-vivo* affinity maturation.^5^

Traditional isolation of antibodies from phage display libraries involves multiple rounds of panning followed by screening and sequencing of positive clones. A key limitation when identifying clones is the screening step following enrichment. Selecting clones can be technically challenging and time consuming, and the process is often hampered by dominant clones within the enriched population, which makes it difficult to identify alternative binders with lower representation within the pool.

Despite recent advances in next-generation sequencing (NGS) technologies, sequence read length is a limitation in their application to phage display screening. The majority of NGS platform technologies allow a maximum read length of up to 500 base pair (bp),^6^ which has extensively been used for the analysis of immunoglobulin (Ig)-repertoires in several species. This length, however, is not sufficient to cover a full single-chain variable fragment (scFv) sequences.^7,8^ Currently, sequencing of the entire variable heavy (VH) and variable light (VL) fragment of a single chain is not routinely carried out and is not possible using conventional NGS platforms. Single-molecule real-time (SMRT) sequencing allows the contiguous sequencing of fragments of up to 8500 bp,^9^ but read length comes at the expense of sequence fidelity.^10^ A recent advance in SMRT sequencing is the addition of hairpin loops to double-stranded DNA fragments.^11^ Consensus sequencing of adaptor ligated fragments can increase the sequence accuracy of an 850 bp fragment (the approximate length of an average scFv) to 99.99%.^12^ Hemadou *et al*. first demonstrated this approach to be effective at accurately identifying full-length scFvs in an enriched fully human, recombinant scFv antibody library.^13^ A separate group has also used it to investigate more in-depth the polyclonal response in phage libraries from simian immunodeficiency virus-infected *Rhesus macaque*.^14^ In both these studies, the analysis was limited to the evaluation of the variable genes repertoire to identify paired VH-VL clones present in their phage display libraries, and no insights on the binding characteristics of the libraries are provided.

Sequencing of the entire scFv in one read identifies paired, functional scFv sequences, without time-consuming screening. Alternative methods of antibody discovery, such as single-cell cloning of antigen-specific B cells, have improved the ability to identify rare antigen specificities. However, these methods are time and resource consuming, and are limited to analysis of a small fraction of the antibody repertoire generated in an animal.^15,16^ Here, we have expanded the combination of phage display panning with SMRT sequencing and open source computational tools to interrogate more in-depth the scFv sequences contained in phage display-selected libraries. This method enables the rapid isolation of functional and specific antibodies from selected phage display libraries, allowing the identification of diverse antibody clones with a broad range of affinities, epitopes and biophysical characteristics.

## Results

### SMRT sequencing illuminates the diversity obtained in a scFv library against CD160 before and after enrichment

**Figure 1.**
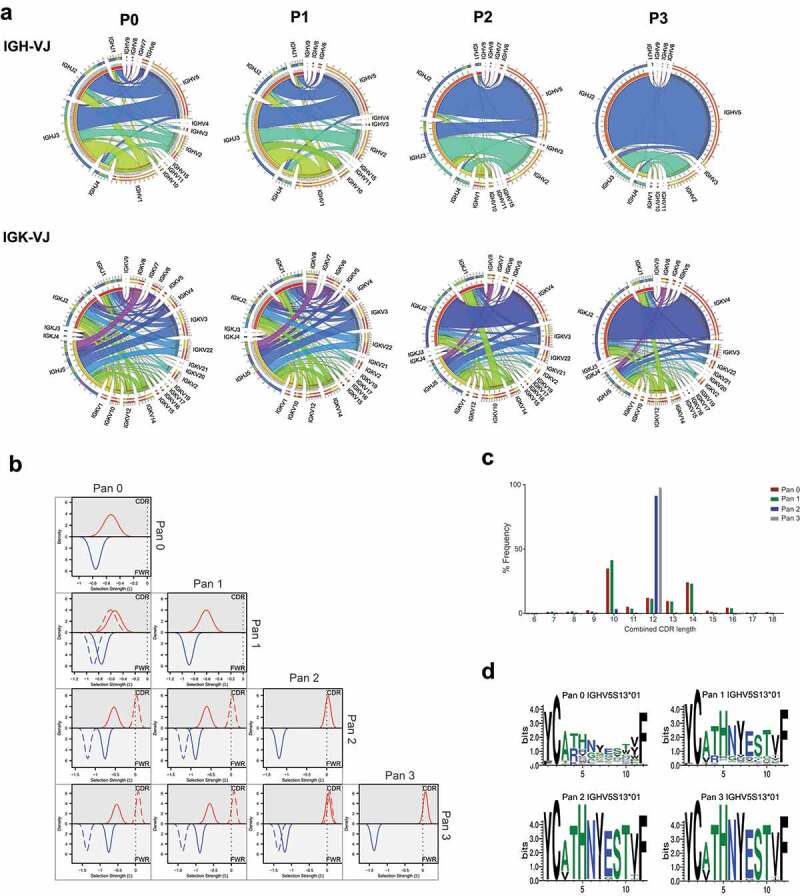
Analysis of CD160 library diversity after phage display enrichment

We analyzed the enrichment of a phage display library against CD160 derived from rats immunized with DNA encoding full-length protein. The library was subjected to three rounds of panning against recombinant antigen. We looked at clonal evolution within the library after each round. Data sets were aligned against the *Rattus norvegicus* germline database.^17^ A total of 9434, 9336, 22660, and 14368 functional reads were obtained from the sequencing of pan 0, 1, 2 and 3, respectively. We investigated the IGHV/IGHJ and IGKV-KJ usage in clones to give an indication of changes in library diversity through rounds of panning. The frequencies of variable and joining regions were visualized using circular correlation analysis (Figure 1a). In pan 0, 519 clones used IGHV1-43 paired with IGHJ2 correlating to 5.5% of the unpanned library. After three rounds of selection, this pairing represented 0.37% of the library, while there was a concomitant increase in the presence of clones using the IGHV5S13 and IGHJ2 gene segments from 2.6% to 60.1%. This demonstrates that the library approached clonality after the third selection round. A similar pattern was observed with kappa chain frequency, where there was an increase from 1.1% to 33% of clones using the pairing IGKV4S9 IGKJ2. A more modest enrichment of IGKV-J pairs is indicative of the ability of antibody heavy chains to pair promiscuously with multiple light chains while retaining function.

[a] Chord diagram representation of the V and J gene frequency and their associations in phage display libraries. Pan 0 represents the unpanned library; pan1, pan2 and pan3 represent the library after the 1st, 2nd and 3rd round of panning, respectively. Correlation plots are shown between IGVH-IGVJ top panel and IGVK-IGVJ bottom panel. [b] Bayesian analysis of highest frequency IGHV genes at each round of panning. Plots are overlaid against analysis from alternative panning rounds to indicate difference in selection distribution. [c] CDR length distribution of all HCDR3 sequences derived from the IGHV5S13*01 germline chain. Amino acids length distribution at the HCDR3 position throughout the panning process. [d] Sequence logo plot of 12 AA length HCDR3 sequences derived from the IGHV5S13*01 germline chain.

To further understand the depth and diversity of sequence, we performed a Bayesian analysis of the VH regions within the library at each round of panning.^18,19^ Mutations across IGHV regions of a group of sequences were compared to the closest parent germline sequence and assigned a selection score Σ. For example, Σ_FWR_ of −1, 0 and 1 indicate strongly negative, neutral, and strongly positive selection, respectively, to the framework regions (FWR) of the sequences (Figure 1b). In pan 0, the highest frequency germline used was IGHV1-43*01, which occurred 945 times (10% of the total sequence). Selection scores of −0.536 and −0.795 were obtained in complementarity-determining region (CDR) and FWR, respectively, suggesting high variability of the library at this stage. At pan 3, analysis of the IGHV5S13 aligned clones demonstrated Σ_CDR_ and Σ_FWR_ of 0.08 and −1.357, respectively (Supplementary Table 1). Thus, at pan 3 the FWR regions show strong evidence of clonality, while there is still variability in the CDRs. This interesting observation suggests a high proportion of the library at pan 3 contains related clones that may represent stages of clonal evolution.

Next, we assessed CDR length distribution within the families. At pan 0 a broad distribution of CDR lengths is observed, and by pan 3 the frequency of clones containing 12 amino acid-length HCDR3 sequences was increased from 18.4% to 97.6% (Figure 1c). The consensus amino acid sequence at these positions clearly showed bias towards a prominent HCDR3, which was already prevalent within antibodies from the same germline before selection (Figure 1d).

### Understanding the prevalence of heavy and light chain pairing during phage display selection

Analysis of heavy and light chain pairing during library generation and selection is not routinely carried out due to sequence length limitations in many NGS platforms. Here we have characterized heavy and light chain pairs through rounds of panning.

**Figure 2.**
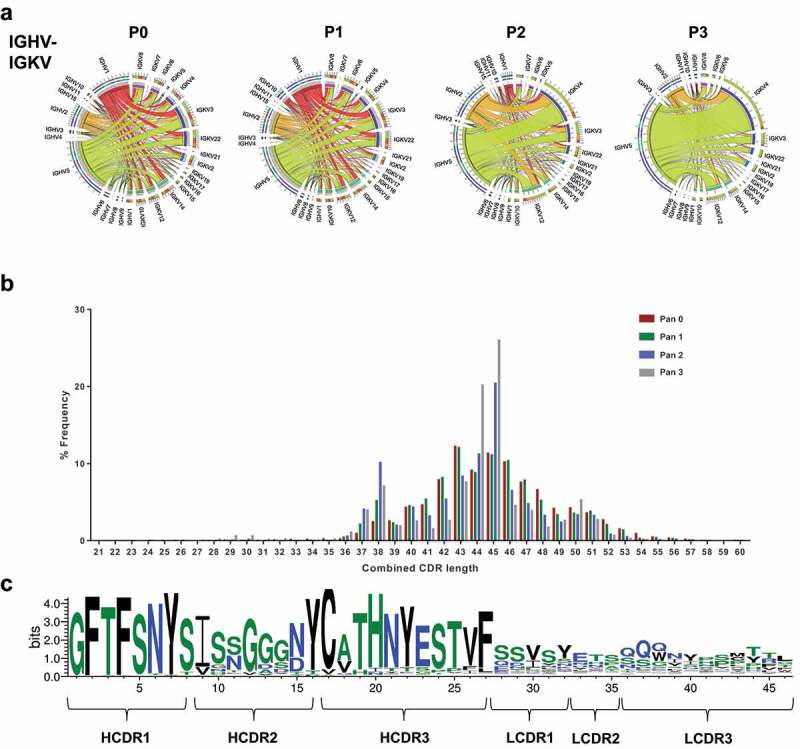
Heavy and light chain pairing in phage libraries

We first investigated the pairing of heavy and light chains and visualized the overall frequency with circular correlation analysis (Figure 2a). As expected, the pairing between heavy and light chains prior to selection was stochastic. The highest heavy-light chain pairing was the combination of IGHV1-43-IGHJ3 with IGKV3S10-IGKJ5, which made up 0.36% of the unpanned library. Unique heavy and light chain combinations constituted 67.74% of the unpanned library. Conversely, at pan 3, 19.5% of the library consisted of clones combining IGHV5S13-IGHJ2 IGKV4S9-IGKJ2 segments and only 16% were unique pairing combinations. The highest frequency heavy and light chains in the library at each round were further represented as a heat map (Supplementary Figure 1). This representation showed that the highly ranked heavy chains paired with many light chains in the unpanned library, but the top-heavy chains only linked to a few specific light chains in pan 2 and 3, highlighting the advantage of sequencing the entire scFv to obtain the correct pairing.

[a] Chord diagram representation of IGVH and IGVK frequency and their associations in phage display libraries. [b] CDR length distribution of all six CDRs from heavy and light chain of antibodies within the CD160 library derived from the IGHV5S13*01-IGVK1 pairs. [c] Sequence logo plot of all six CDRs from the heavy and light chain of antibodies within the preselected CD160 library derived from the IGHV5S13*01-IGVK1 pairs.

### Somatic mutation in library sequences

We sought to examine somatic mutations across the entire scFv by analyzing the sequence across the six CDRs. The combined CDR length distribution showed an increase in antibodies with a peak combined CDR length of 46 amino acids as the selection progressed (Figure 2b). The sequence logo analysis for the antibodies with the same combined length highlighted a canonical CDR structure for the highest represented IGHV-IGHK pairing. The same HCDR3 sequence observed in the heavy chain only analysis demonstrated high diversity across the CDRs in the light chain in addition to diversity in HCDR2 (Figure 2c). We further looked at the variability in amino acid sequence across the six CDRs in the top 10 most abundant heavy and light chains families observed in the library after selection (Supplementary Figure 2). Variability was observed primarily in HCDR2, suggesting the selection had enriched clones from the same germline with somatic mutations.

### Identification, generation, and biophysical characterization of clones based on NGS data analysis

**Figure 3.**
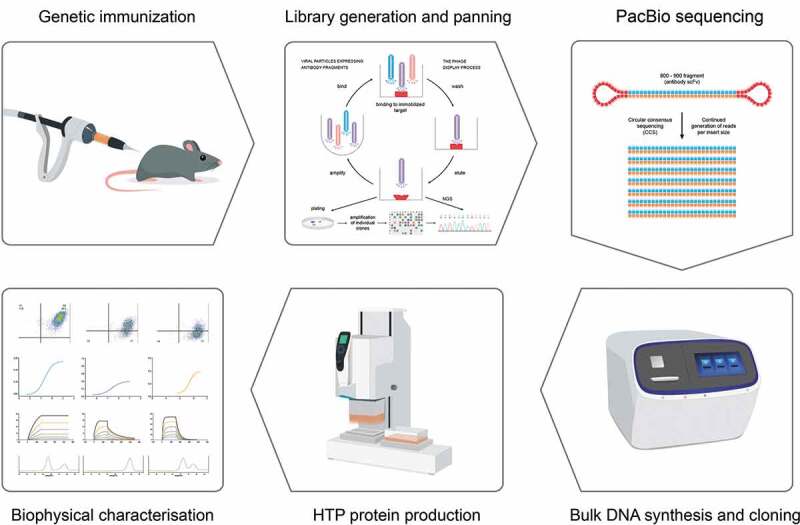
Overview of strategy to generate antibodies from deep-sequenced scFv libraries

We sought to determine the quality and diversity of CD160-binding antibodies obtained through NGS analysis. We devised a simple workflow for the identification and testing of scFv antibodies (Figure 3). Initially, to identify binders, we based our analysis on the frequency of heavy chains and further searched for unrelated clones based on unique IGHV usage. The strategy was applied to data from pan 3 of the CD160 library. Here, we sorted for antibody clonotypes, defined as clones with identical HCDR3 sequence. Within each clonotype family several variants existed with substitutions in HCDRs 1 and 2. A single clone within each clonotype family was selected, based on the distance of HCDRs 1 and 2 from germline, for further characterization. In previous experiments we identified 5 clonotypes through conventional phage display selection and screening of over 200 phage clones.^20^ All clonotypes found through conventional screening methods were observed in the NGS data set.

Animals are immunized by genetic vaccination. Phage display libraries are derived from successfully seroconverted animals and subjected to 2–3 rounds of panning followed by sequencing using PacBio SMRTbell technology. Hairpin adapters are ligated to each fragment of DNA, providing a binding site on the chip and a uniform priming site for the DNA polymerase. The polymerase reaction resembles a rolling circle replication and creates multiple reads of each molecule, which are used to calculate a consensus sequence. Clones selected from analyzed sequences are generated using DNA printing technology and produced using high throughput expression prior to biophysical analysis.

**Figure 4.**
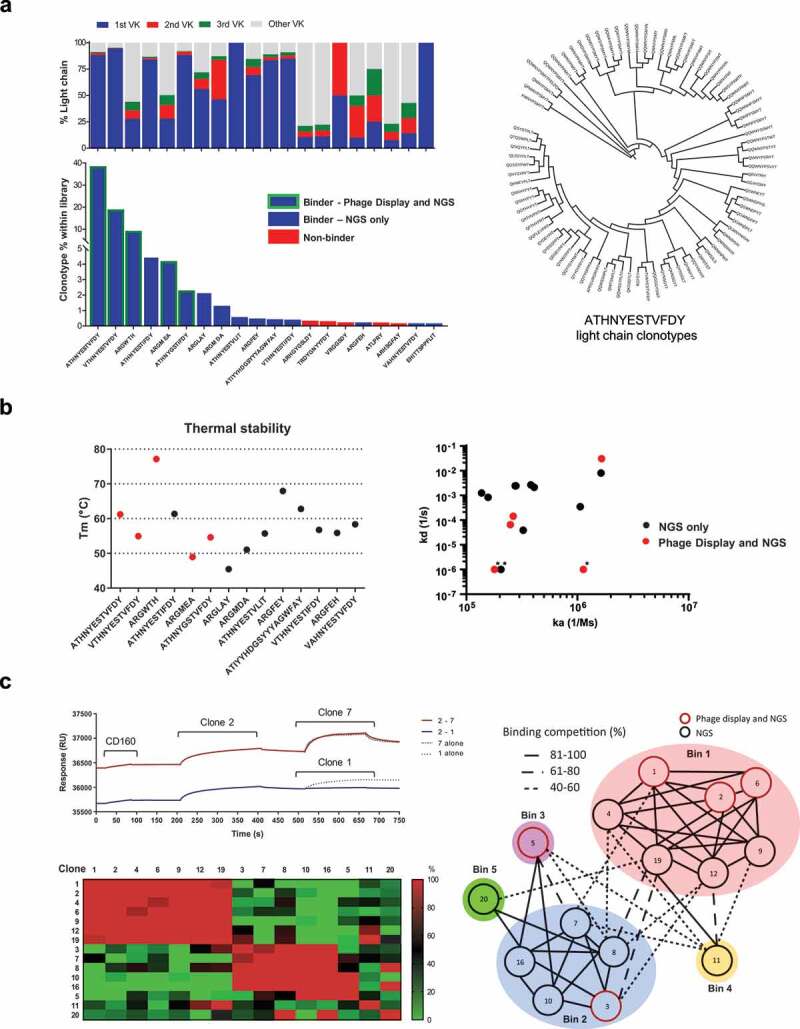
Identification and characterization of scFv clones

We observed that for each HCDR3 clonotype family, between 1 and 77 kappa variable (VK) clonotypes were present (Figure 4). We carried out functional testing on antibodies using the top ranked IGVK clonotype by frequency for each HCDR3.

[a] Bottom panel, flow cytometry analysis of scFv antibodies based on HCDR3 sequence of the top 20 most abundant HCDR3 sequences, 15 were shown to bind by flow cytometry (blue bars), 5 of which were identified also by phage display (green border). Top panel, relative abundance of IGVK clonotypes for each HCDR3 sequence. The top 3 IGVK clones are highlighted in blue, red and green, additional VK clonotypes grouped in grey. Right panel, circular phylogenetic tree of the IGVK clonotypes identified for the most represented HCDR3. [b] Left panel, differential scanning fluorimetry analysis of antibodies obtained from NGS analysis of enriched CD160 library. Binders identified also via phage display are marked in red. A range of melting temperatures were obtained, spanning from 45°C to 78°C. Right panel, Biacore SPR affinity measurement of selected HCDR3 clonotypes for recombinant CD160. Measured on rates (ka 1/Ms) ranging from 1.37 × 10^5^ to 1.65 × 10^6^ and off rates (kd 1/s) ranging from 3.05 × 10^–2^ to below 1 × 10^–6^. Binders additionally identified via phage display are marked in red. * = kd restricted to 1 × 10^–6^ (1/s). [c] Epitope binning experiments using CD160 binding scFvs. Representative competition sensorgrams are shown from two bins, along with heat map of relative binding competition of antibody pairs. A total of five distinct epitope bins were identified. Phage selected HCDR3 clonotypes marked in red circles on epitope bin representation.

The top 20 clones were placed into scFv-Fc format and screened by flow cytometry; from these, 15 demonstrated binding to cells expressing CD160 in flow cytometry screening and 5 had previously been identified through conventional screening (Figure 4a). The thermal stability of the derived antibodies was next investigated using differential scanning fluorimetry (Figure 4b). The stability (Tm) of the scFv portion of the different antibodies ranged from 45–78^o^C. Of the 15 identified binders, one scFv (EHITTSPPFLIT) did not exhibit typical thermal denaturation properties despite the specific binding on CD160. Binding kinetics of the scFv antibodies to recombinant CD160 were determined by surface plasmon resonance (SPR) (Supplementary Figure 3). Antibodies with binding kinetics in the pM to nM range were obtained, with a broad range of kinetic profiles summarized in Table 1. Clone 1, 3 and 12 had unmeasurable off rates in this experiment and were artificially limited to 1 × 10^–6^ (1/s) to enable comparison. Taken together, the clones identified solely by NGS demonstrated comparable biophysical properties to the clones derived by traditional phage display selection. The most represented 5 clones from the NGS sequence analysis were further tested in a poly-reactivity ELISA assay against an array of diverse antigens,^21^ showing minimal non-specific interactions for one of the scFv tested (Supplementary Figure 4). The scFvs obtained were grouped into epitope bins (Figure 4c). The antibodies fell into one of 5 unique bins. Antibodies obtained through conventional phage screening largely clustered into one epitope bin, with two antibodies in a second and third independent bin. Antibodies obtained through NGS analysis demonstrated binding to two further unique epitopes.

**Table 1.** Summary of the top 20 CD160 scFv identified

Clone	HCDR3	VH gene	P3%	Obtained in conventional screening	ka (1/Ms)	kd (1/s)	KD (M)
1	ATHNYESTVFDY	IGHV5S13	38.6	Yes	1.79E+05	<1E-06*	<5.58E-12*
2	VTHNYESTVFDY	IGHV5S13	19	Yes	2.49E+05	6.51E-05	2.62E-10
3	ARGWTH	IGHV2-70	9.3	Yes	1.13E+06	<1E-06*	<0.89E-12*
4	ATHNYESTIFDY	IGHV5S13	4.5	No	3.25E+05	3.90E-05	1.20E-10
5	ARGMEA	IGHV2-70	4.2	Yes	1.65E+06	3.05E-02	1.84E-08
6	ATHNYGSTIFDY	IGHV5S13	2.3	Yes	2.64E+05	1.43E-04	5.41E-10
7	ARGLAY	IGHV2-70	2.2	No	1.06E+06	3.50E-04	3.30E-10
8	ARGMDA	IGHV2-70	1.3	No	1.63E+06	8.04E-03	4.92E-09
9	ATHNYESTVLIT	IGHV5S13	0.58	No	4.08E+05	2.11E-03	5.18E-09
10	ARGFEY	IGHV2-70	0.48	No	2.75E+05	2.45E-03	8.91E-09
11	ATIYYHDGSYYYAGWFAY	IGHV5S13	0.43	No	1.37E+05	1.25E-03	9.15E-09
12	VTHNYESTIFDY	IGHV5S13	0.41	No	2.05E+05	<1E-06*	<4.88E-12*
13	ARHGYGSLDY	IGHV5S13	0.31	No	-	-	ND
14	TRDYGNYYFDY	IGHV6-8	0.24	No	-	-	ND
15	VRGGSDY	IGHV5S13	0.23	No	-	-	ND
16	ARGFEH	IGHV2-70	0.23	No	2.79E+05	2.43E-03	8.72E-09
17	ATLPRY	IGHV5S13	0.18	No	-	-	ND
18	ARHSGFAY	IGHV5-25	0.18	No	-	-	ND
19	VAHNYESTVFDY	IGHV5S13	0.18	No	1.57E+05	8.33E-04	5.31E-09
20	EHITTSPPFLIT	IGHV5S13	0.1	No	3.81E+05**	2.66E-03**	6.98E-09**

Biophysical properties of the top 20 CD160 identified binders, by HCDR3 frequency, after 3 rounds of panning. (P3% = frequency of the HCDR3 in the round 3 of biopanning, ka = on-rate, kd = off-rate, KD = affinity). Clones which did not demonstrate binding in flow cytometry experiments are highlighted in grey (ND = not determined).

*kd outside instrument limits. kd value restricted to 1E-06.

**Outside instrument limits

### Mining NGS data to obtain binders with altered biophysical properties

**Figure 5.**
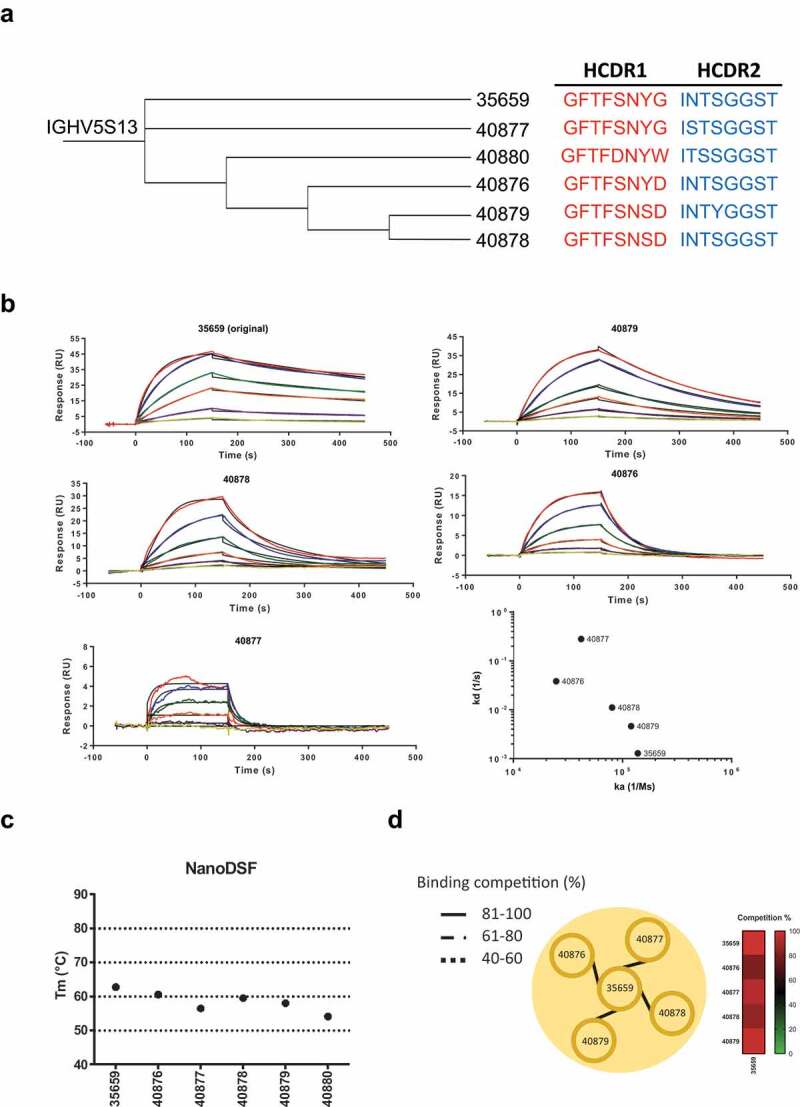
Identification of alternative binders in the NGS data set

NGS data analysis identified several clones with identical HCDR3 sequences but containing mutations in HCDR1 and HCDR2. As per our previous analysis, we maintained a single light chain for each HCDR3 clone based on the highest frequency pair identified after PacBio sequencing. We chose clones with a greater distance from the germline sequences of HCDRs 1 and 2 for characterization. We hypothesized that clones with alternative HCDR1 and HCDR2 sequences represented diverse antibodies at various stages of affinity maturation, and thus attempted to generate a panel of antibodies with unique binding kinetics based on a conserved binding epitope. Starting from an antibody clone with high thermal stability in our top 20 clones (ATIYYHDGSYYYAGWFAY), we identified alternative sequences containing the same HCDR3 but associated with different HCDRs 1 and 2. Sequences were compared to the identified germline antibody sequence (Figure 5a). The scFv antibodies that were generated bound with a range of affinities, from the micromolar to nanomolar range (Figure 5b). Differences in affinity were mainly driven by a change in the dissociation rate of the antibody, which varied 2 Logs (1.29 × 10^−3^ to 2.82 × 10^–1^). Amongst the modified scFv antibodies, clone 40880 lost the capability to bind, the substitution of G for W in CDR1 is clearly not accepted in the binding interface and was the furthest from germline sequence. It is tempting to speculate that this might represent an unproductive step in the clonal evolution of this antibody, but the possibility of sequencing errors cannot be discounted. Importantly, all of the antibodies generated maintained high thermal stability, and, as expected, competed for the same epitope on the protein (Figure 5c).

[a] Phylogenetic analysis of CD160 library from pan 3 showing HCDR1 (red) and HCDR2 (blue) variants related to clone 35659 (clone 11 – ATIYYHDGSYYYAGWFAY) and diversion from germline sequence. [b] Biacore SPR binding data demonstrating the range of affinities for clone 35659 and relatives, targeting the same epitope on CD160. Right panel, on rates (ka 1/Ms) and off rates (kd 1/s) plot ranging from 2.47 × 10^4^ to 1.38 × 10^5^ and 1.29 × 10^–3^ to 2.82 × 10^−1^, respectively. [c] Left panel, thermal stability of clone 35659 HCDR1-HCDR2 sequence relatives, with Tm ranging from 54.2°C to 62.8°C. Right panel, epitope binning experiment on 35659 related clones showing high competition and single epitope bin association.

### Identification of alternative binding in highly clonal libraries

We applied PacBio sequencing technology to a second library generated from rats immunized with the target antigen CD123. CD123 is a cell surface receptor that transmits signal from the cytokine interleukin 3 and has been identified as a possible target in the treatment of acute myeloid leukemia.^22,23^ The library was chosen for analysis because the generation of antibodies using standard clone selection and expression had yielded few unique hits. Of 200 screened clones, only 6 unique clones that showed specificity toward CD123-positive SupT1s by flow cytometry were identified. We analyzed the clonal distribution of the CD123 library after three rounds of selection. A total of 5193 IGH and 1361 IGK clonotypes were identified. IGHV1 and IGHV5 were observed to be the most abundant v-genes, with pairing to IGHJ3 and IGHJ2 predominantly favored. Again, V gene utilization for IGK was more diverse, with IGKV4 (20%), IGKV22 (13%), IGKV3 (11%), IGKV10 (11%), IGKV12 (9%), IGKV14 (8%), IGKV8 (7%), IGKV6 (6%) and IGKV2 (4%) all present within the library. IGKJ1 (44%) was the most abundant, closely followed by IGKJ5 (36%) and IGKJ2 (20%) (Supplementary Figure 5a). Analysis of IGHV-IGKV pairing showed that IGHV1 appeared to favor pairing with IGKV12 (60%), while IGHV5 favored pairing with IGKV4 (50%) following enrichment.

In order to select antibodies for synthesis and testing, candidate HCDR3 sequences were again chosen based on library abundance. In this instance, however, closely related sequences were avoided to increase the diversity of selected candidates (Supplementary Figure 5b). A total of 37 IGH clonotypes were selected. Each heavy chain clonotype was also expressed with three candidate IGK sequences, excluding the clones derived from phage display manual selection. Clones were transiently expressed and tested for binding to cells expressing CD123. Flow cytometry staining showed CD123-specific binding with 30 of 37 selected clonotypes (Supplementary Figure 5c). For 12 of the 24 NGS-selected clonotypes, we observed that all 3 selected IGVK chains were functional in the context of the scFv, in 5 cases 2 IGVK chains were functional and in the remaining 7 a single functional IGVK chain was observed. In 16 of the 24 cases, we observed that the most prevalent VK chain generated a functional scFv, further supporting the strategy previously used of selecting the highest represented light chain within the heavy chain clonotypes.

## Discussion

NGS technologies have been used in combination with phage display in a number of applications.^24,25^ While NGS allows in-depth analysis of phage display libraries, its widespread use has been hampered by limitations in read length^26,27^ and signal integrity^16^ of several sequencing technologies. These limitations have meant that most sequencing applications have only covered a single variable domain within a library, showing the greatest utility in the sequencing of single domain antibody libraries.^28,29^ Yang and colleagues recently described the use of phage display data obtained using MiSeq sequencing to isolate antibodies from scFv libraries that had undergone multiple rounds of panning. The data sets obtained had high sequence depth and allowed generation of antibodies against the target antigen of interest; however, a key limitation to the method was the use of limited read length within the sequencing data that required additional PCR amplification to retrieve the full sequence of the clones.^30^ Here, we used the PacBio sequencing platform combined with SMRTbell technology to generate highly accurate full-length scFv sequence repertoires from immune libraries selected through conventional phage display biopanning targeting the cell surface antigens CD160 and CD123. Mining these two independent libraries enabled the identification of a large and diverse panel of high-quality antibodies.

Hemadou *et al*. demonstrated that the combination of SMRT sequencing with circular consensus adapter ligation allowed the identification of full-length scFvs from an enriched phage display library. By tracking a manually selected clone, they identified its presence in the top 100 of the pool of reads and they were able to determine an accuracy of >99.9% for complete sequences of inserts approaching 1,000 bp. Although this supports the robustness of the combinatorial method, it does not give insights on the functionality and diversity of the scFvs contained in the phage library.^13^ Similarly, Han and colleagues used this approach to investigate phage display libraries that had shown high clonality using traditional screening methods and, although they identified several new clones, they did not investigate the specificity and functionality of the phage display-selected antibodies.^14^

In our study, we used long-read sequencing to expand the screening of selected phage display libraries. This combination not only provides additional knowledge on the antibody repertoire of immunized animals but greatly enhances the screening power of immune phage display libraries for the identification of functional and diverse scFv fragments. As 5% of the immunoglobulin repertoire in rodents expresses the lambda light chain, we have considered the kappa chain locus only.^31^ We analyzed data from two immune libraries against CD160 and CD123 based on HCDR3 abundance within the enriched libraries, and selected clones either based on frequency or phylogenetic distance to allow the isolation of more diverse binders. An interesting observation was that not all clones enriched by phage display panning bound to target antigen, but we observed a success rate of 75% and 81% when we analyzed the most frequent clones prevalent after CD160 and CD123 selection, respectively. This strategy consists of conventional phage display biopanning followed by SMRT sequencing and open source NGS analysis to identify an expansive repertoire of antigen-specific antibody clones, with virtually no screening required.

A disadvantage of the system deployed here, relative to second-generation systems such as the Illumina platform, is the far lower sequencing depth obtained. Typically yields of 80,000 reads for a 1 Kb sequence fragment can be generated, as compared to over 5 × 10^6^ reads for Illumina Hiseq sequencing. Clearly, the read depths obtained in our experiments were insufficient to cover the theoretical diversity of a typical phage display library. It is possible to modify the sequencing protocol by sequencing the same prepared library on multiple SMRT cells of the sequencer. As library preparation and instrument run time are a substantial proportion of the sequencing cost, this approach could enable sequencing depth to be increased 10-fold with a modest increase in sequencing costs. For the purpose of this study and using an approach in which libraries are first enriched against target antigen, we surmise that depth of sequencing obtained in a single run is sufficient to sample our immune scFv phage display libraries, which typically have reduced diversity and clonal redundancy. This is clearly the case for libraries after multiple rounds of enrichment. However, for libraries on which limited enrichment have been performed or where a broader view of an immune repertoire is desired, the depth of sequencing may become limiting.

Increasingly, antibody discovery has become a high throughput empiric endeavor. Antibodies with desired properties, such as receptor agonism or the appropriate binding kinetics, within a population may be rare. Thus, it is often necessary to sample highly diverse populations in order to obtain the antibody of interest. Our library sequencing approach allows the isolation of large numbers of antibodies that have been affinity matured *in-vivo* and are thus likely to retain excellent specificity and biophysical properties.^32^

Single cell screening technologies such as SLAM^33^ and microfluidic-based^34^ platforms similarly offer an excellent source of *in-vivo* matured antibodies, but themselves suffer limitations. In B cell binding strategies, screening of the antibody repertoire often requires live cells to be manipulated and sorted for antigen binding, limiting the antibody repertoire to memory B cells that express surface IgG. Microfluidic-based platforms allow the real-time interrogation of plasma cells that secrete IgG, but these technologies are technically complex and require specialized instrumentation. The strategy that we describe is relatively low cost, simple and easily adoptable by most labs while offering a depth and diversity of antigen specific antibodies similar to single cell methods. Single B cell methods have a clear advantage in the native pairing of heavy and light chains, but they are limited to a small numbers of antigen-specific B cell that can be efficiently sorted and recovered before the sequence identification.^15,35^

Here we have described a simple method using SMRT sequencing to interrogate diversity from enriched phage display libraries to identify antigen-specific scFvs. The interrogation of the full-length sequences of each clone provides the advantageous information of functionally paired VH-VL selected during the phage display biopanning rounds. A logical next step for this technology will be to incorporate techniques such as single cell emulsion PCR^36^ at the library generation stage. Combining these methods will enable the generation and full screening of natively paired heavy and light chain libraries, thus offering all the advantages of phage display and B-cell screening methods.

## Materials and methods

### Library construction and bio-panning

Three Wistar rats were immunized using genetic vaccinations with DNA encoding for CD160 and CD123, the DNA plasmids of interest were coated on gold nanoparticles and administered intramuscularly using a GeneGun™ (Biorad). Vaccinations were carried out at Aldevron, GmBH, Freiburg. Following vaccination, total RNA was extracted from the spleen of animals using an RNAeasy™ (QIAGEN) extraction kit. RNA obtained from multiple animals was reverse transcribed to cDNA, which was used to generate phage display libraries cloned into the phagemid vector *pHEN1*.^37^ Libraries were generated as previously described with a size of 1 × 10^6^ and 1 × 10^8^ for CD160 and CD123, respectively.^20^ Bio panning was carried out on Strep-Tactin® (IBA) magnetic beads using in-house generated proteins that incorporated a streptagII tag for direct capture from transfected HEK293T cells. After three rounds of biopanning, antigen binding tests from individual bacterial colonies were performed as previously described.^20^

### PacBio SMRTbell™ library assembly

DNA from various libraries was amplified using primers specific for the phagemid vector *pHEN1* backbone (M13 Rev caggaaacagctatgac and M13 Fd3 gtcgtctttccagacgttagt).

PCR amplification was carried out on 1 ug of plasmid DNA using 20 cycles of amplification to reduce the risk of PCR bias. PCR products were purified using a Mini amp Kit (QIAGEN) according to the manufacturer’s instructions. The amplified DNA libraries were prepared following the PacBio guidelines and sequenced on an SMRT cell using Pacific Biosciences RS sequencing technology (Pacific Biosciences) at GATC GMBH (GATC). Input DNA quality and concentrations were measured using a Qubit Fluorometer dsDNA Broad Range assay (Life Technologies, p/n 32850). The SMRT bell was produced using the DNA Template Prep Kit 1.0 (Pacific Biosciences; p/n 100-259-100). A Bioanalyzer 2100 12 K DNA Chip assay (Agilent Technologies, p/n 5067-1508) was used to assess the fragment size distribution. A blunt end ligation reaction followed by exonuclease treatment was performed to create the SMRTbell template.

### NGS analysis, ranking and clone selection

The selected libraries were quality inspected and quantified on the Agilent Bioanalyzer. Ready-to-sequence SMRTbell-polymerase Complex was created using the P6 DNA/Polymerase binding kit 2.0 (Pacific Biosciences, p/n 100-236-500) according to the manufacturer’s instructions. The Pacific Biosciences RS2 instrument was programed to load and sequence the sample on a single SMRT cell v3.0 (Pacific Biosciences, p/n100-171-800), taking one movie of 120 min. The MagBead loading method (Pacific Biosciences, p/n 100–133-600) was chosen in order to improve enrichment of the longer fragments. At the end of the run, a sequencing report was generated for every cell, via the SMRT portal. Thereby, the adapter dimer contamination, the sample loading efficiency, the obtained average read length and the number of filtered sub-reads were assessed.

ScFv libraries were sequenced by PacBio sequencing. A total of 15395, 10044, 26802 and 19499 reads were generated from biopanning rounds 0, 1, 2 and 3, respectively. Total reads were filtered to contain between 900 and 1200 bp fragments that include phage PelB leader sequence and myc tag to obtain full-length sequences. These were then processed using one of two methods: sequence was either analyzed using IMGT/HighV-QUEST^17^ or by a custom pipeline to obtain in frame paired antibody heavy and light chains. The resulting functional reads represented 61.3%, 93.0%, 84.5% and 73.7%, for each of the biopanning round dataset.

In the custom pipeline, the linker sequence was used to identify binders, long read sequences were first split into VH and VL sequences and each were annotated using a standalone version of IgBlastn.^38^ The rat immunoglobin germline gene sequences from the international ImMunoGeneTics (IMGT) database (http://www.imgt.org) were used for numbering and annotation. Each VL sequence was annotated by IGK genes, and the final light annotation was designated as that with the higher identity to the corresponding V gene. The alignments from IgBLAST were processed and summarized by Change-O,^39^ which provides a tabular output for all CDR elements to enable ranking.

Sequences were further processed with ClustalOmega-1.2.2 using a full distance matrix and the Kimmura correction. The intermittent tree files were visualized using Interactive Tree Of Life (iTOL) v3 (https://itol.embl.de/about.cgi).^40^

Bayesian analysis of immunoglobulin sequences was carried out using Baseline v1.3 (http://selection.med.yale.edu/baseline). A focused selection was used as the statistical framework. FWR/CDR boundaries were defined based on IMGT numbering.

### Generation of scFv constructs

ScFv antibody sequences were generated through DNA synthesis. DNA sequences, as retrieved from the phage display library, were synthesized as gBlocks (Integrated DNA Technologies) with flanking restriction sites *AgeI* and *BamHI*. The scFv fragments were cloned into a modified version of the expression vector AbVec2.0^41^ containing an in-frame mIgG2a Fc region.

### Surface plasmon resonance

SPR experiments were performed with a Biacore T200 instrument using HBS-P^+^ as the running and dilution buffer (GE Healthcare, p/n BR100671). Biacore Insight Evaluation software v3.0 (GE Healthcare) was used for data processing. For determination of binding kinetics, goat anti-mouse IgG (GE Healthcare, p/n BR100838) was covalently coupled to a CM5 Sensor Chip (GE Healthcare, p/n 29149603) according to manufacturer recommendations (approximately 9000 RU). ScFvs with a murine IgG2a Fc were captured (level range 200–400 RU), and concentrations of interaction partner protein from 200 nM with 2-fold serial dilutions, were injected over the flow cell at a flow rate of 30 µl/minute. A double reference subtraction was performed using buffer alone. Kinetic rate constants were obtained by curve fitting according to a 1:1 Langmuir binding model. KD for clones 1, 3 and 12 were calculated using an artificially limited kd of 1 × 10^–6^ (1/s) due to instrument limitations.

### Differential scanning fluorimetry

Protein stability was analyzed on a Prometheus NT.48 (NanoTemper). The emission of fluorescent radiation with the wavelengths of 330 nm and 350 nm was measured with the temperature changes from 20°C to 95°C, at a rate of increase of 1°C min^−1^. The fluorescence curves obtained were differentiated, and the ratio of differentials was used to determine the melting temperature of the proteins.

### Cross-reactivity ELISA

The ELISA was performed in-house using a standard set of six antigens used to test antibodies cross-reactivity.^21^ Human Insulin (Sigma, p/n I9278-5 ML), dsDNA (Sigma, p/n D4522-1 MG), ssDNA (Sigma, p/n D8899-1 MG), Cardiolipin (Sigma, p/n C0563-10 MG), LPS (Invivogen, p/n tlrl-eblps) and KLH (Sigma, p/n H8283-50 MG) were coated at 1 μg/ml in phosphate-buffered saline (PBS) on Nunc MaxiSorp plates. After 2% bovine serum albumin block step, the top 5 anti-CD160 clones in scFv-murine IgG2a Fc format were used as primary antibodies at 1 µg/ml in duplicate and compared to specific positive control antibodies for each antigen (anti-insulin Abcam, p/n ab8304; anti-dsDNA Abcam, p/n ab27156; anti-ssDNA and cross-reactive for Cardiolipin, Sigma, p/n MAB3868; anti-LPS Abcam, p/n ab35654; anti-KLH Abcam, p/n ab34607). Plates were washed 4 times in PBS 0.05% Tween20, to remove unbound antibodies, prior to incubation with secondary anti-mouse IgG-HRP antibody (Jackson Laboratories, p/n 115-005-062). Positive interaction was revealed using 1-Step Ultra TMB substrate (ThermoFisher Scientific, p/n 34028). Reaction was stopped with a solution of 1 M sulfuric acid and absorbance read at 450 nm using Multiskan plate reader (ThermoFisher Scientific).

### Flow cytometry

SupT1 cells were engineered to overexpress the human CD160 or PSMA. Cloned scFv antibodies were used as the primary antibody for staining 1 × 10^5^ antigen positive supT1 cells followed by 1 µl of anti-mouse secondary antibody (Jackson ImmunoResearch, p/n 115-116-146). Cells were acquired on a MACSQuant analyzer flow cytometer.

### Epitope binning

The assay was performed with a Biacore T200 instrument using HBS-P^+^ as the running and dilution buffer (GE Healthcare, p/n BR100671). BIAevaluation software version 3.0 (GE Healthcare) was used for data processing. Soluble recombinant His-tagged CD160 (Sino Biological, p/n 12191-H08H) was captured on a NiNTA Series S chip at 0.5 µg/ml and then saturated with a first anti-CD160 antibody at 150 nM concentration. To determine binding competition, a second anti-CD160 antibody (150 nM) was injected over the flow cells. Percentage of binding competition for the second antibody was determined by normalization of same antibody pair to adjust for dissociation, and then compared with the Rmax obtained in the absence of the first competing antibody.

### In silico analysis

3D structure of heavy and light chain variable domains was obtained via structure prediction and homology modeling on BioLuminate 3.8 (Schrodinger). Surface charge analysis was performed via the Protein Surface Analyzer tool on BioLuminate 3.8 (Schrodinger). Area for charge and hydrophobic patches was calculated for the regions encompassing the CDR loops.

## Supplementary Material

Supplemental Material

## Abbreviations

Ig: immunoglobulin
CDR: complementarity-determining region
FWR: framework region
NGS: next-generation sequencing
scFv: Single-chain variable fragment
SMRT: single molecule real-time
SPR: surface plasmon resonance
Tm: melting temperature
VH: variable heavy
VK: variable kappa
VL: variable light
PBS: phosphate-buffered saline
BSA: bovine serum albumin
