# Supplementary material for: Combining phage display with SMRTbell next-generation sequencing for the rapid discovery of functional scFv fragments
Source: MAbs. 2020 Dec 31;13(1):1864084. doi: 10.1080/19420862.2020.1864084 (PMC7781620; doi:10.1080/19420862.2020.1864084)

a

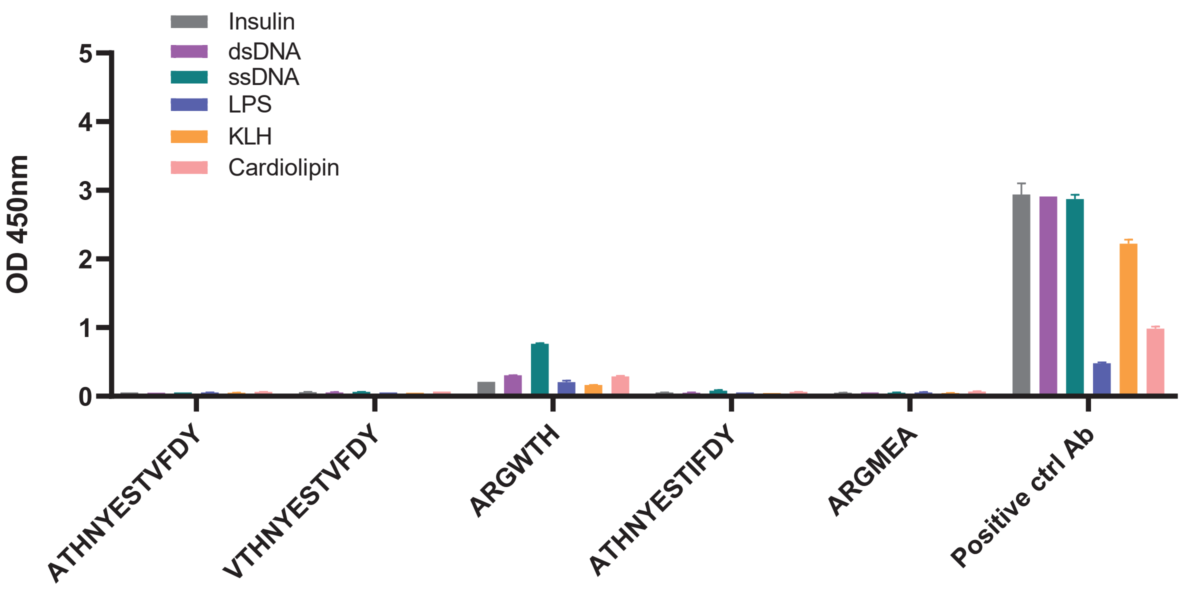

b

Clone	CDR Positive charge area ($Å^2$)	CDR Negative charge area ($Å^2$)	CDR Hydrophobic area ($Å^2$)	Total Charge	Sum AggScore
ATHNYESTVFDY	316.8	550.8	613	3.00eV	88.53
VTHNYESTVFDY	570.9	540.1	417.3	3.00eV	85.21
ARGWTH	54.1	1147	677.6	-1.00eV	88.9
ATHNYESTIFDY	424.6	592.2	317	3.00eV	91.76
ARGMEA	270.2	522.1	331	3.00eV	14.11

c

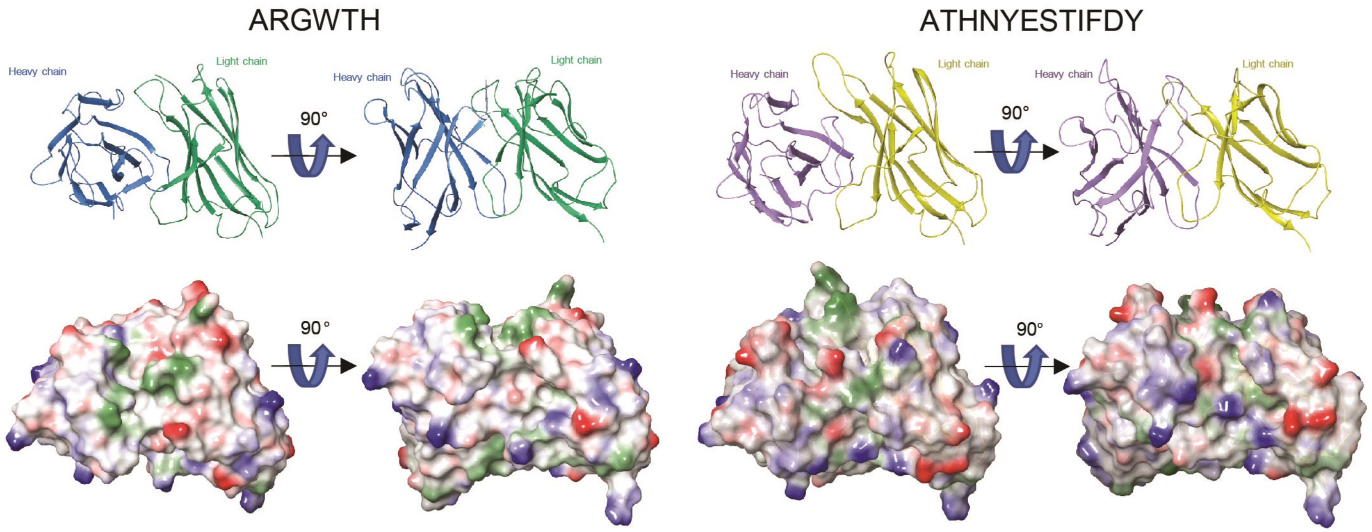

Supplement: Supplemental Material [file KMAB_A_1864084_SM1738.zip › supplement/Supplementary_figure_4_reduced.pdf]
